# Metabolite Signatures in Hydrophilic Extracts of Mouse Lungs Exposed to Cigarette Smoke Revealed by ^1^H NMR Metabolomics Investigation

**DOI:** 10.4172/2153-0769.1000143

**Published:** 2015-05-12

**Authors:** Hu JZ, Wang X, Feng J, Robertson BJ, Waters KM, Tilton SC, Pounds JG, Corley RA, Liu M, Hu M

**Affiliations:** 1 Pacific Northwest National Laboratory, Richland, WA, USA; 2 State Key Laboratory of Magnetic Resonance and Atomic and Molecular Physics, Wuhan Institute of Physics and Mathematics, the Chinese Academy of Sciences, Wuhan, China

**Keywords:** ^1^H-NMR metabolomics, Mouse lungs, Cigarette smoke, PCA, OPLS, Obesity

## Abstract

^1^H-NMR metabolomics was used to investigate the changes of metabolites in the lungs of mice with and without being exposed to a controlled amount of cigarette smoke. It was found that the concentrations of adenosine derivatives (i.e. ATP, ADP and AMP), inosine and uridine were significantly changed in the lungs of mice exposed to cigarette smoke when compared with controls regardless the mice were obese or of regular weight. The decreased ATP, ADP, AMP and elevated inosine suggested that the deaminases in charge of adenosine derivatives to inosine derivatives conversion would be significantly changed in the lungs of mice exposed to cigarette smoke. Indeed, transcriptional study confirmed that the concentrations of adenosine monophosphate deaminase 2 and adenosine deaminase 2 were significantly changed in the lungs of mice exposed to cigarette smoke. We also found that the ratio of glycerophosphocholine (GPC) to phosphocholine (PC) was significantly increased in the lungs of obese mice compared with those of the regular weight mice. The GPC/PC ratio was further elevated in the lungs of obese group exposed to cigarette smoke.

## Introduction

Exposure to cigarette smoke is one of the major risk factors inducing pulmonary and bronchial injury either through direct toxic effects or indirectly by initiating inflammatory responses [1]. The adverse effects on the immune system of cigarette smoke exposure compromise the host's ability to stimulate appropriate immune and inflammatory responses and may persist for decades after exposure has ended [2]. The inhaled cigarette smoke alters host-microorganism interaction dynamics and causes inflammations that trigger the development of chronic respiratory diseases [3,4]. Smoke increases the risk of infections, including structural changes in the respiratory tract such as mucosal permeability and decrease in immune response, and is a substantial risk factor for important bacterial and viral infections, which is believed to pivotal to progressions of cancer, heart disease, and chronic obstructive pulmonary disease (COPD), etc [5,6]. It is estimated that there will be 10 million premature deaths worldwide per year by 2030 attributed to smoking [7].

Obesity is a metabolic disorder associating with the development of metabolic syndrome, including hyperlipidemia, insulin resistance and type 2 diabetes, that affects one third of adults and one fifth of children in the United States, as well as 300 million adults worldwide according to the World Health Organization's report [7,8]. There is increasing evidence that obesity enhances tumor development [9], and is associated with cancers in breast, kidney, thyroid, etc., according to the National Cancer Institute (NCI).

Previous studies indicated that smoking and obesity showed synergetic effects [10], where the authors analyzed the association between smoking and body mass index with no molecular information pursued. The co-occurrence of smoking and obesity may lead to progression of atherosclerosis and therefore is of primary concern for public health [11]. However, the molecular mechanisms underlying the impact of smoking and the synergetic effects of smoking and obesity are poorly understood. Metabolites, the intermediate and end products of a metabolic pathway or multiple pathways, are likely to be important for understanding the molecular mechanisms. Although there are a few studies on the impact of metabolite signatures in the lungs either by smoking, or by obesity [12,13], there is no systematic investigation on the metabolites in the lungs of both the regular weight and the obese animal models that are exposed to a controlled amount of cigarette smoke.

Metabolomics, the study of low molecular weight molecules or metabolites found in cells and biological systems [14-17], has emerged as an important new tool in elucidating the molecular mechanisms and pathways in biological systems using, e.g., biofluids, tissues and organs. Nuclear Magnetic Resonance (NMR) spectroscopy is one of the leading analytical tools for metabolomics research [18-21]. ^1^H NMR is especially attractive because proton is present in virtually all metabolites and its NMR sensitivity is high, enabling the simultaneous identification and monitoring of a wide range of metabolites with concentration above, e.g., ∼1.0 μM. To our knowledge, nearly all investigations evaluating the impact of cigarette smoke on human or mouse lungs were based on genomics or proteomics [22-24]. The first study using the metabolomics approach to assess effects of mainstream cigarette smoke on human lung epithelial cells was conducted by Suryanarayana in 2009 [12]. Another metabolomics-based work exploring tobacco-related global metabolome in blood was published in 2013 [25]. However, none of these previous studies had ever directly investigated the adverse influence of cigarette smoke on lung tissue.

In this work, high resolution liquid state ^1^H-NMR spectroscopy was employed for global metabolic profiling of excised lungs from mice of both regular and obese body weight with and without being exposed to a controlled amount of cigarette smoke. Multivariate data analysis, both principal component analysis (PCA) and orthogonal projections to latent structures (OPLS), were carried out for pattern recognition and for identifying metabolite signatures that differentiate groups. To our knowledge, this is the first time that the effects of cigarette smoke to metabolites, and the synergetic effects between smoking and obesity in the lungs of mice of both regular and obese body weight, are investigated by the metabolomics approach.

## Methods

### Chemicals

3-(trimethylsilyl)-2,2′,3,3′-tetradeuteropropionate (TSP-d_4_) and Sodium azide (NaN_3_) were purchased from Sigma-Aldrich, Ltd. (Missouri, USA). Deuterated chloroform (CDCl_3_) containing 0.03% (v/v) tetramethylsilane (TMS) was obtained from Alfa Aesar, A Johnson Matthey Company (Massachusetts, USA). Methanal and chloroform were purchased from Fisher Scientific, Inc. (New Hampshire, USA). Deuterium oxide (D_2_O, 99.9% in D) was from Cambridge Isotope Laboratories, Inc. (Miami, USA).

### Animal experiments and sample collection

A total of 32 C57BL6 mice of 13 weeks in age, consisting of 16 non-obese (RW) and 16 diet-induced-obesity obese (OB) mice, were purchased from Jackson Laboratories (Bar Harbor, ME) and were housed at Pacific Northwest National Laboratory (PNNL)'s animal facility and were acclimated for one week before the exposure experiments. Each of the RW and OB groups was randomized into two sub-groups with n=8, either subjected to sham exposure or cigarette smoke exposure to be detailed below. Cigarettes (25,000 sticks of 3R4F reference cigarettes) were purchased from University of Kentucky (Lexington, KY). All cigarettes were conditioned to ISO standard 3402 (ISO, 1991b) at 22 ± 1°C and 60 ± 3% RH before use for smoke generation. The Jaeger-Baumgartner 2070i cigarette-smoking machine (JB2070 CSM; CH Technologies, Westwood, NJ) was used to generate mainstream smoke. A mainstream puff was drawn from the cigarette by negative pressure airflow maintained through the siphon port by a pump operating at a flow rate of ∼1.05 L/min. To achieve the target concentration in the exposure unit, the main smoke (MS) flow was diluted with clean, humidified air to make up the total inlet flow required to provide atmosphere for the exposure chamber. Groups of mice with or without obesity (∼15-weeks old at start of exposures) were exposed to either filtered air (sham controls, SC) or mainstream (MS cigarette smoke) by nose-only inhalation exposure for 5 h/day for a total of eight exposures over two weeks as follows: 5 consecutive days of exposure, followed by 2 days with no exposure, then three days of exposure, with necropsies occurring the day following the last exposure. Target cigarette smoke exposure concentration was 250 μg wet-weight total particulate matter (WTPM)/L of air for the MS exposures (8 mice / group).

At the time of sample collection, mice were sacrificed using a 70/30 CO_2_/O_2_ mix. Inhalation of 70/30 CO_2_/O_2_ mix utilized the equipment provided by the Vivarium according to their standard operating protocol. This method is consistent with the recommendations of the Panel on Euthanasia of the American Veterinary Medical Association. Immediately after sacrificing the animal, right lung was obtained and snap frozen in liquid nitrogen and then stored at -80 °C until used for metabolomics study. Modified Folch method was adopted for tissue extraction by following the published protocol [21]. Briefly, tissue samples from the four different groups were randomly extracted by ice-cold MeOH-CHCl_3_-H_2_O (i.e. using a mixture of 250 μl methanol, 250 μl chloroform and 175 μl water for 30-40 mg tissue). Randomization of all the samples and establishment of an extraction sequence of samples by randomly assigning an extraction priority for each sample is important for suppressing systematic errors that could be introduced at different times. Step-1: After being removed from the -80°C refrigerator, the lung tissue was quickly weighed (1minute) and then homogenized using a Tissue Tearor (Model 985-370, BioSpec Products, Inc.) after adding 4 ml MeOH and 0.85 ml H_2_O per gram of tissue placed inside a glass vial with outside surrounded by an ice bath (1 minute), followed by vortexing the mixture (2 minutes) and then adding 2 ml chloroform per gram of tissue, vortexing again (2 minutes). At the end of the step-1, the enzymes should have been adequately destroyed. Step-2, 2 ml chloroform and 2 ml H_2_O per gram of tissue was added in the mixture followed by vortexing again (2 Minutes), transferring the two layers into two clean new glass vials separately with syringes. Finally the solvents were removed either by lyophilizer for the MeOH/H_2_O layer, i.e. the hydrophilic extracts, or by nitrogen gas flow dry for the CHCl_3_ layer, i.e. the hydrophobic extracts. The extracts were then stored at -80°C until NMR measurements. Special attention was paid to make sure that the sample extraction procedures, in particular the timing of the first step, were kept identical for each sample so that sample degradation, if any, was identical between the samples.

### ^1^H NMR spectroscopy of tissue extracts

Shortly before the ^1^H NMR experiments, the hydrophilic extracts were reconstituted in 600 μl of D_2_O containing 0.5 mM TSP-d_4_. About 550 μl of the prepared sample was loaded into a standard 5 mm NMR tube (Wilmad, Buena, NJ) inside a cold room at 5°C. To prevent biodegradation, 0.2% sodium azide (w/v) was added into the solution. The NMR experiments were carried out on a Varian 600 MHz spectrometer equipped with a Z axis-gradient 5 mm HCN probe. All the NMR measurements were carried out at 25°C. The standard Varian PRESAT pulse sequence using a single pulse excitation and 0.5 s low power pre-saturation at the H_2_O peak position for H_2_O suppression was used for the measurements. For acquiring each spectrum, an accumulation number of 1024 scans with acquisition time of 1s covering a spectral width of 16 ppm and recycle delay time of 3.5 s were used.

### NMR data pre-processing and multivariate data analysis

All free induction decays were multiplied by an exponential function with 0.5 Hz Lorentz line broadening prior to Fourier transformation. And, all ^1^H NMR spectra were manually phased and baseline corrected using the Processor module of Chenomx (NMR suite 7.6, Professional) and referenced to the chemical shift of TSP-d4 at 0 ppm. For hydrophilic extracts, the spectral regions at δ 0.5 ∼ 9.0 were segmented into discrete bins with equal width of 0.004 ppm using the Profiler module of Chenomx. Spectral regions at δ 3.34 ∼ 3.38 and δ 4.7 ∼ 5.1, containing MeOH and residual water signals, respectively, were excluded from analysis. The integral areas of all bins in this study were first divided by the area of TSP-d4 whose concentration was constant across the sample studied and then normalized to per unit weight of lung tissue measured before extraction. Then, the normalized NMR data in terms of spectral bins was imported into SIMCA (Version 13.0.3 64-bit, Umetrics, Umea, Sweden) for multivariate data analysis, i.e. principal component analysis (PCA) and orthogonal projection to latent structure (OPLS). PCA was performed first using the mean-centered and unit-variance scaled NMR data to obtain an overview and detect possible outliers. Subsequently, OPLS was conducted using the autoscaled data as ***X***-matrix (with each row representing a sample, each column representing a binned chemical shift range) and class information as ***Y***-matrix to find significant variables, i.e. metabolites, responsible for discrimination of the two different classes. Y = 0 was assigned to the control group and y = 1 to the experimental group before building an OPLS model, so that positive loadings mean up-regulated while negative loadings mean down-regulated. Both PCA and OPLS models were constructed using the non-linear iterative partial least squares (NIPALS) algorithm and model complexity (number of components) was determined by a 7-fold cross-validation method. Model quality can be evaluated from parameters such as R^2^, revealing the interpretability of the model, and Q^2^, indicating the predictability of the model. Finally, the model significance was further assessed by the CV-ANOVA test at the level of *p*<0.05. S plot (named S-line plot in SIMCA-13 for NMR spectral data), a useful visualization tool for interpretation of multivariate classification, was employed to help identify statistically significant metabolites and therefore potential biomarkers. In this plot, loadings obtained from the OPLS model were plotted with color-coded correlation coefficients denoting the variable importance for class separation with warm colored (e.g. red) metabolites being more significant than cold colored (e.g. blue) ones. A cutoff value, depending on sample number in each group, was chosen to select metabolites responsible for between group variations based on the discrimination significance (*p*<0.05).

The animal protocol was approved by PNNL's Institutional Animal Care and Use Committee (IACUC). The methods were carried out in accordance with the approved guidelines.

## Results

### ^1^H NMR spectra of lung tissue extracts

**Figure 1:**
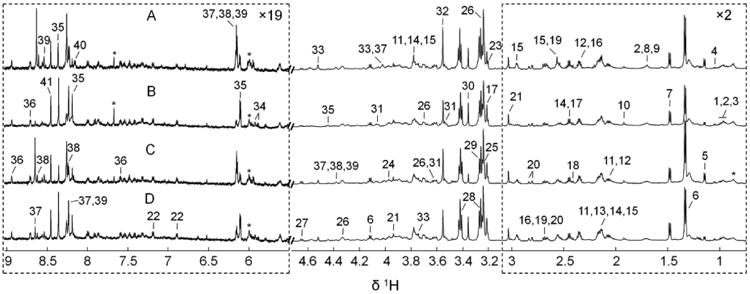
600 MHz liquid state ^1^H NMR spectra of the hydrophilic extracts of lungs excised from the cigarette smoke exposed and the control mice. A: Regular weight sham control (RW-SC), i.e. non-obese and non-smoke. B: Regular weight mainstream smoke (RW-MS), i.e. non-obese smoke exposed. C: Obese sham control (OB-SC). D: Obese mainstream smoke (OB-MS). The peak intensities were normalized to per unit weight of lung tissue before extraction. The dotted regions were vertically expanded 19 (left side part) and 2 (right side part) times, respectively. “*Asterisks*” indicated unassigned peaks. A total of 41 metabolites were identified with metabolite numbers, i.e. the metabolite keys shown in Table 1.

Examples of typical ^1^H NMR spectra of hydrophilic extracts obtained from both the cigarette smoke exposed and the control groups were shown in Figure 1. Peak intensities were normalized to per unit weight of lung tissue before extraction, thus the concentrations of a given metabolite in the four different groups can be directly compared visually according to the peak intensities in the corresponding spectrum. Peak assignments were listed in Table 1. A total of 41 metabolites were identified using Chenomx and the chemical shift identities of metabolites were assigned according to both literatures [26-30] and the Chenomx metabolite library. Due to spectra complexity including heavy overlap of resonances from small molecules and broad peak features from residual lipoproteins, and the relatively low signal-to-noise ratio of aromatic spectral region as well as quantity limitations of Chenomx metabolite library, deconvolution of the whole spectrum was only applied to selected samples for peak assignment purpose and qualitative analysis, i.e. an untargeted metabolomics approach, was employed in this study. Spectral deconvolution of a representative sample was shown in Figure S1 in Supporting Information. A wide range of amino acids, carbohydrates, glycolysis and tricarboxylic acid cycle (TCA cycle) intermediates were detected. Other observed metabolites included choline metabolites, ethanolamine metabolites, purine and pyrimidine derivatives. Visual inspection of the ^1^H NMR spectra revealed apparent spectral signature differences among these randomly selected representatives. For example, spectrum B from a RW-MS (regular weight, mainstream smoke) mouse had lower levels of ATP (peak 38), ADP (peak 39) and AMP (peak 37) as shown in Figure 1B, compared with spectrum A from a RW-SC (regular weight, sham control) mouse shown in Figure 1A, while the cigarette smoke exposed mouse showed higher levels of uridine (peak 34) and inosine (peak 35). Similar spectral features were observed when spectrum D from an OB-MS (obesity, mainstream smoke) mouse was compared with spectrum C from an OB-SC (obesity, sham control) mouse. To identify significantly changed metabolites across different groups, i.e. taking within-group variations into consideration, in the following both PCA and OPLS were performed on the above spectra set (the RW-SC group, the RW-MS group, the OB-SC group, and the OB-MS group). The spectra of hydrophobic extracts of mouse lung tissues were not displayed, since no statistical metabolic variation was observed between the treated group and the control group.

### Cigarette smoke and/or obesity induced metabolic alterations in lung tissue extracts

**Figure 2:**
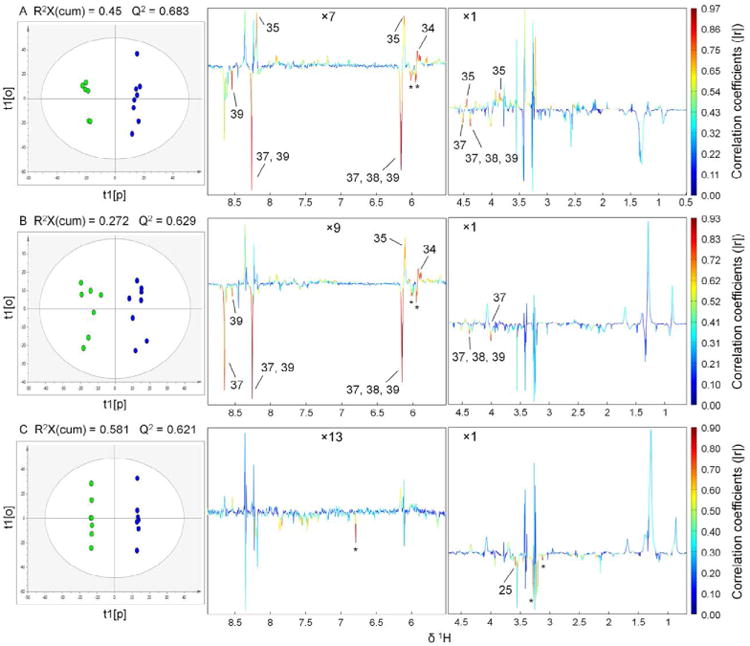
OPLS scores (left) and coefficients-coded loadings plot (right) of the model discriminating the control (green dots) and the experimental (blue dots) groups: A, RW-SC (control) *vs* RW-MS (experimental); B, OB-SC (control) *vs* OB-MS (experimental); C, RW-MS (control) *vs* OB-MS (experimental). Other model parameters: A, R^2^(cum) = 0.99, R^2^Y(cum) = 1; B, R^2^(cum) = 0.945, R^2^Y(cum) = 1; C, R^2^(cum) = 1, R^2^Y(cum) = 1. CV-ANOVA results gave *p* values of 0.059, 0.029 and 0.348 for models A, B and C, respectively. Regions were vertically expanded as denoted in the figure. “*Asterisks*” indicated unassigned peaks. Metabolite keys were shown in Table 1.

Unsupervised (or exploratory) data analysis, i.e. PCA in this case, was conducted firstly to get an overview of the ^1^H NMR spectral binning data set of hydrophilic extracts and detect possible outliers. Multivariate data analysis were conducted between four pairs of groups, i.e. RW-SC *vs* RW-MS, OB-SC *vs* OB-MS, RW-SC *vs* OB-SC, RW-MS *vs* OB-MS. PCA scores plots were shown in Figure S2 in Supporting Information. Outliers detected from PCA analysis were excluded from further OPLS modeling. In order to maximize the correlation between ***X***-matrix (NMR spectra data set) and ***Y***-matrix (the class information) as well as the variation in ***X***-matrix, OPLS was performed to assess variable importance and determine discriminatory variables (i.e. metabolites) responsible for separation of different groups [31]. Values of the resulting model parameters, i.e. R^2^ and Q^2^, showed good quality of the generated OPLS models (Figure 2A-C). The OPLS model validities were further assessed by the CV-ANOVA analysis [32]. The key variables showing significant differences between the control group and the experimental group were extracted from the correlation coefficients-coded loadings plots of the OPLS models. Note that only three OPLS models were successfully constructed and the results were summarized in Figure 2. For OPLS analysis, y = 0 was assigned to the control group in each OPLS model, i.e. RW-SC in model A, OB-SC in model B, RW-MS in model C, and y = 1 was assigned to the experimental group, i.e. RW-MS in model A, OB-MS in model B, OB-MS in model C, to build the corresponding OPLS model. Construction of OPLS model failed for RW-SC *vs* OB-SC using spectra data of hydrophilic extracts even after exclusion of the outliers detected in Figure S1D, indicating that the RW-SC and the OB-SC groups cannot be separated from each other when hydrophilic metabolites were concerned. As shown in Figure 2, the control groups were well discriminated from the corresponding experimental groups for the three successful cases. After excluding the outliers due to bad water suppression, OPLS resulted in cutoff values of |r| > 0.755, |r| > 0.707, |r| > 0.707 for model A, model B and model C in Figure 2, respectively, for correlation coefficients as significant based on the discrimination significance (*p*<0.05) [29,33,34]. Metabolites showing significant differences between the control groups and the corresponding experimental groups in Figures 2A-2C and therefore responsible for the classification of different groups were extracted from the coefficients color-coded loadings plots with their correlation coefficients tabulated in Table 2. It was known from Figure 2A and Table 2 that the concentrations of inosine and uridine were significantly elevated while the concentrations of adenosine derivatives, i.e. ATP, ADP and AMP, were significantly decreased in the RW-MS group. As shown in Figure 2A, two unassigned peaks at δ = 6.02 and δ = 5.95 were selected as discriminatory variables between the RW-SC group and the RW-MS group with correlation coefficients of -0.880 and -0.881, respectively. These unassigned peaks may be resonances of some pyrimidine metabolites such as uridine diphosphate glucuronate (UDP-glucuronate) and uridine diphosphate galactose (UDP-galactose) according to our previous publication [30], but cannot be assigned with confidence in this study because of the relatively low signal-to-noise ratio in the corresponding spectral regions. The same results were obtained from Figure 2B and Table 2 in selecting the discriminatory metabolites between the OB-SC group and the OB-MS group, indicating that cigarette smoke exposure can cause similar major metabolic alterations in the lung tissue regardless whether the mice were obese or of regular body weight. Since the extraction procedure was carried out in ice bath, i.e. tissue samples were not freeze clamped in liquid nitrogen during extraction, ATP, ADP and AMP levels observed may be variable due to degradation of ATP to produce ADP and AMP [35]. However, since tissue samples from the four different groups were randomly extracted following the same strategy and analyzed by NMR spectroscopy, measurement errors maybe potentially introduced by ATP depletion were averaged out across the whole sample set. Besides, suppose the errors caused by non-freeze-clamping, ATP levels will be decreased while ADP and AMP levels will be increased. However, levels of ATP, ADP, and AMP observed in this study were all significantly decreased in the smoke group compared with the non-smoke group whether the mice were obese or not, indicating that cigarette smoke indeed caused statistical metabolic signature alterations in the hydrophilic extracts of mice lung tissues. Figure 2C showed the metabolic pattern differences between the RW-MS group and the OB-MS group, i.e. metabolic phenotype differences of cigarette smoke exposed mice induced by obesity. We found that phosphocholine and three unassigned peaks at δ = 6.79, δ = 3.28 and δ = 3.12 were significantly down-regulated in the OB-MS group in Figure 2C with correlation coefficients of -0.896, -0.897, -0.855 and -0.864, respectively. However, CV-ANOVA test of model C in Figure 2 gave a *p* value of 0.348, suggesting that the corresponding OPLS model was not statistically significant, further discussion of the results obtained from Figure 2C was therefore omitted. But researchers can still benefit from model C and special attention should be paid to phosphocholine and the other three unassigned peaks marked in Figure 2C if investigations of obesity induced lung damage of cigarette smoke exposed population compared with non-obese population using large sample size are of interest.

Stimulated by the tentative findings from Model C, where phosphocholine maybe of importance in the lungs for evaluating the risk of obesity in cigarette smoke exposed group and the fact that the glycerophosphocholine/phosphocholine ratio, i.e. GPC/PC ratio, has been proposed as a bio-indicator of several types of cancer [36-38], we evaluated the levels of GPC/PC ratios in four different groups to investigate influence of obesity and cigarette smoke exposure on the GPC/PC ratio using two way ANOVA [39]. GPC/PC ratios and the two way ANOVA design table were shown in Table S1 in Supporting Information. The two factors, i.e. obesity and cigarette smoke exposure, were assigned as class type 1 and class type 2, respectively, as shown in Table S1. Replicates in each specific combination of class type 1 and class type 2 were listed in Table S1 after excluding outliers, i.e. 6, 7, 7, and 7 samples for the RW-SC group, the RW-MS group, the OB-SC group, and the OB-MS group, respectively. Results of the two way ANOVA for GPC/PC ratio were listed in Table 3. As shown in Table 3, total variation of the GPC/PC ratio across the four different groups was divided into two parts, i.e. within group variance and between group variance. The between group variance was composed of three subparts, including variation between the two different groups of class type 1 (variance caused by obesity), variation between the two different groups of class type 2 (variance caused by cigarette smoke exposure), and the interaction between class type 1 and class type 2 (synergy between obesity and cigarette smoke exposure). Significance of the effects of these terms on GPC/PC ratio can be evaluated from the *p* values listed in Table 3. It was concluded from Table 3 that obesity can significantly influence GPC/PC ratio in hydrophilic extracts of mouse lung tissues while cigarette smoke exposure caused no statistical alterations of GPC/PC ratio. Back checking the GPC/PC ratios listed in Table S1 revealed significantly elevated levels of GPC/PC ratio induced by obesity, where the mean values of GPC/PC ratio were 4.06 for the RW-SC group, 4.44 for the RW-MS group, 6.00 for the OB-SC group and 7.07 for the OB-MS group, respectively. Additionally, the interaction term in Table 3 also had significant impact on GPC/PC ratio, indicating synergetic effect between obesity and cigarette smoke exposure. Interestingly, obesity and cigarette smoke exposure alone only accounted for 43.91% and 3.62%, respectively, of the between group variance, while the synergy between obesity and cigarette smoke exposure took much greater responsibility for the between group variance (52.47%). This was further confirmed by the corresponding *p* values listed in Table 3. These results suggested that the obese population may be more subject to lung damage than the non-obese population if exposed to cigarette smoke.

**Figure 3:**
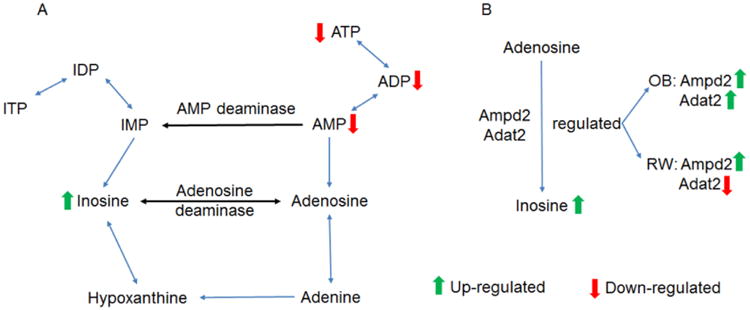
A: Part of the purine metabolism pathway as depicted in the Small Molecule Pathway Database (SMPDB) (http://www.smpdb.ca/); B: The detailed changes of enzymes from the parallel transcriptomics analysis shown in Figure 4. AMP: adenosine monophosphate; ADP: adenosine diphosphate; ATP: adenosine triphosphate; IMP: inosine monophosphate; IDP: inosine diphosphate; ITP: inosine triphosphate; OB: diet-induced-obese (i.e. the OB-SC group and the OB-MS group); RW: regular weight (i.e. the RW-SC group and the RW-MS group); Ampd2: Adenosine monophosphate deaminase 2; Adat2: Adenosine deaminase, tRNA-specific 2.

**Figure 4:**
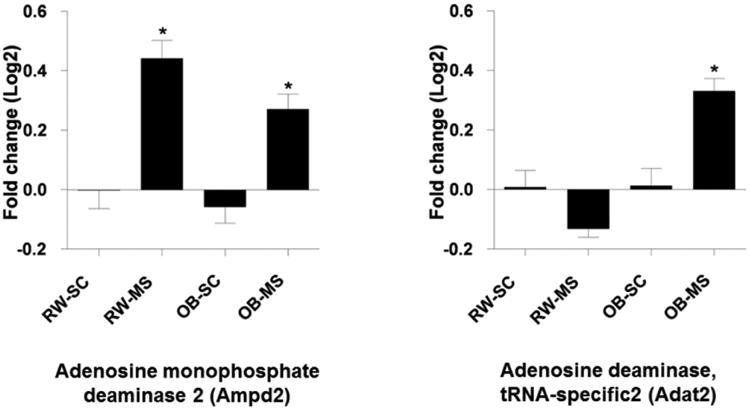
Alterations of the purine metabolism pathway related enzymes revealed by the parallel transcriptomics study. OB: diet-induced-obese (i.e. the OB groups discussed earlier). “*Asterisks*” indicated *p* < 0.05 compared to RW-SC animals.

From the above statistical analysis results on metabolites, it was found that cigarette smoke exposure can cause significant changes of adenosine derivatives, i.e. ATP, ADP, AMP, and inosine in mouse lung tissue extracts. This finding indicated that the molecular pathway related to adenosine and inosine derivatives must be altered in lung tissues of the mice due to cigarette smoke exposure, i.e. the purine metabolism pathway must be disturbed. As depicted in the Small Molecule Pathway Database (SMPDB) (http://www.smpdb.ca/) [40], in the purine metabolism pathway, inosine, adenosine derivatives and their related enzymes are key pathway components (Figure 3). In this pathway, the enzymes of AMP deaminase (Ampd) is in charge of the inter conversion of AMP (adenosine monophosphate) to IMP (inosine monophosphate) while the enzyme of adenosine deaminase (Adapt) is responsible for the inter conversion of adenosine to inosine. The significantly altered levels of inosine and adenosine derivatives in lung tissues observed in this study suggested that the concentration of deaminases (either AMP or adenosine deaminase, or both) must be significantly changed as a result of lung injury due to cigarette smoke exposure. We have previously carried out comprehensive transcriptomics investigation on the left lungs (while the right lungs were for NMR metabolomics studies) from the same mice of this study and the detailed transcriptional response of the mouse lung to cigarette smoke exposure with and without obesity can be found in our previous publication [41], where a total number of 3012 pulmonary genes were found differentially expressed across the study at a significant level of *p* < 0.01 with a 5% false discovery rate. Stimulated by the metabolomics findings from this work, we re-evaluated the trascriptomics data by focusing on the purine metabolism pathway, we found that the concentrations corresponding to adenosine deaminase (Adat2) and adenosine monophosphate deaminase 2 (Ampd2) were significantly altered in the lungs of mice exposed to smoke. Specifically, we found that adenosine deaminase (Adat2) was slightly down-regulated while adenosine monophosphate deaminase 2 (Ampd2) was up-regulated in lung tissue of non-obese mice (the RW-MS group) after exposure to cigarette smoke (Figure 4). For the obese mice, it's interesting that both Ampd2 and Adat2 were elevated after smoke exposure (the OB-MS group) compared with the control (Figure 4). This again suggested that the impact of obesity and cigarette smoke exposure were different in the obese group compared with the regular weight group, echoing the finding above from GPC/PC ratio evaluations. Despite this difference, both metabolomics and transcriptomics results showed consistent results that the purine metabolism was altered and the meanings of this change will be further discussed below.

## Discussion

The decreased ATP, ADP, AMP and elevated inosine found in this study predicted that the deaminases in charge of adenosine derivatives to inosine derivatives conversion were altered in the lungs of mice exposed to cigarette smoke. Our previous transcriptomics study from the same project showed that in the lungs of non-obese animals, the transcripts for adenosine monophosphate deaminase 2 (Ampd2) were up-regulated and adenosine deaminase (Adat2) were slightly down-regulated after smoke exposure compared with the sham controls. And in the groups with diet-induced-obesity (OB), both adenosine monophosphate deaminase 2 (Ampd2) and adenosine deaminase (Adat2) were up-regulated. Despite the difference of Adat2 between the regular weight and the obese groups, which also indicated obesity played a somewhat different role in modulate the purine metabolism pathway compared with regular weight mouse, both metabolomics and transcriptomics studies showed that the purine metabolism was fluctuated, indicating that adenosine signaling pathway played important role in inflammatory responses. Furthermore, the GPC/PC ratio was significantly increased in lungs of the obese mice compared with that of the regular weight mice. The GPC/PC ratio was further significantly elevated in the lungs of the obese group by cigarette smoke exposure.

ATP, ADP and AMP are phosphorylation derivatives of adenosine which is considered a mediator of a variety of physiological processes including respiratory regulation, neural function, lymphocyte differentiation and many other metabolic distresses [42]. The observed reduction of adenosine derivatives, i.e. ATP, ADP, AMP and elevation of inosine in mice with both regular body weight and obese body weight in this study clearly pointed out that adenosine signaling pathway was profoundly perturbed by cigarette smoke exposure. Adenosine derivatives play many important biological roles in addition to being components of DNA and RNA [43]. For example, it has been proposed that adenosine derivatives accumulate through adenosine signaling in recruiting neutrophils in lungs, and are then detoxified by adenosine deaminases to inosine derivatives, which exert anti-inflammatory effects [44-47]. The activity of adenosine deaminase is associated with increasing hydrolysis of adenosine monophosphate (AMP) to compensate platelet aggregation in rats exposed to cigarette smoke [48], and curcumin could modulate this purinergic signaling by regulating the thrombus formation as an antioxidant [49].

For the both up-regulated adenosine monophosphate deaminase 2 (Ampd2) and adenosine deaminase (Adat2) in groups with obesity, a recent review suggested that there may be a closer interaction between the inflammatory events and obesity [50], indicating possible different mechanisms in obese mouse compared to the acute lung injury observed after smoke exposure in regular weight mice. However, as shown in Figure 2C, although the OB-MS group and the RW-MS can be well separated in the OPLS scores plot, CV-ANOVA test of model C in Figure 2 gives poor validity of the model suggesting that larger sample size is required to make the model performance reliable. Like adenosine derivatives, uridine has shown anti-inflammatory effects and appears to affect the tumour necrosis factor (TNF, also known as TNF-alpha) levels in lungs [51]. Elevated level of uridine and decreased level of unassigned peaks of some pyrimidine metabolites observed in this study suggested that cigarette smoke exposure can cause perturbation of pyrimidine metabolism. Inosine has been reported to have immunomodulatory and neuroprotective effects in animal models of sepsis, ischemia-reperfusion and autoimmunity [52].

Higher levels of GPC/PC ratio may suggest the possibility of an alteration in glycerophosphodiesterase, such as Endometrial Differential 3 (EDI-3), which regulates the choline metabolism [36]. It has become increasingly clear that high GPC concentration is associated with poor prognosis in breast cancer and elevated GPC/PC level is an established indicator of triple-negative breast cancers [53], while a decreased GPC/PC ratio has been reported in ovarian and prostate cancers compared with normal tissue [37]. In this work, we found that the GPC/PC ratio was significantly increased in the lungs of obese mice compared with that of regular weight mice. Cigarette smoke exposure alone cannot induce significant alteration to GPC/PC ratio in hydrophilic extracts of murine lung tissues. Further, we found that the synergetic effect between cigarette smoke exposure and obesity showed significant impact on GPC/PC ratio. As GPC/PC ratio is a known bio-indicator of several cancer types, the results suggest that the obese population may have a statistically higher chance of developing lung disease compared with the non-obese population when exposed to cigarette smoke [54,55].

## Supplementary Material

supporting information

## Figures and Tables

**Table 1:** Peak assignments of metabolites in lung tissue extracts.

Key	Metabolites	δ ^1^H (ppm) and multiplicity^a^
1	Isoleucine	0.94(t), 0.99(d), 1.25(m), 1.46(m), 1.97(m), 3.66(d)
2	Leucine	0.96(d), 0.97(d), 1.67(m), 1.70(m), 1.73(m), 3.73(m)
3	Valine	0.97(d), 1.02(d), 2.26(m), 3.60(d)
4	Isobutyrate	1.05(d), 2.38(m)
5	Propylene glycol	1.14(d), 3.43(dd), 3.54(dd), 3.87(m)
6	Lactate	1.33(d), 4.12(q)
7	Alanine	1.48(d), 3.78(q)
8	Lysine	1.42(m), 1.50(m), 1.70(m), 1.89(m), 1.93(m), 3.02(t), 3.75(t)
9	Arginine	1.63(m), 1.72(m), 1.87(m), 1.93(m), 3.24(t), 3.77(t)
10	Acetate	1.92(s)
11	Homoserine	2.05(m), 2.16(m), 3.77(m), 3.85(dd)
12	Glutamate	2.06(m), 2.12(m), 2.33(m), 2.37(m), 3.75(dd)
13	Methionine	2.12(m), 2.14(s), 2.19(m), 2.65(t), 3.85(dd)
14	Glutamine	2.13(m), 2.15(m), 2.43(m), 2.47(m), 3.78(t)
15	Glutathione	2.16(m), 2.18(m), 2.53(m), 2.58(m), 2.94(dd), 2.97(dd), 3.76(d), 3.78(m), 4.58(m)
16	Malate	2.36(dd), 2.68(dd), 4.30(m)
17	Carnitine	2.42(dd), 2.46(dd), 3.21(s), 3.40(m), 3.41(m), 4.56(m)
18	Succinate	2.41(s)
19	Citrate	2.53(d), 2.69(d)
20	Aspartate	2.68(dd), 2.82(dd), 3.89(dd)
21	Creatine	3.03(s), 3.94(s)
22	Tyrosine	3.04(dd), 3.19(dd), 3.93(dd), 6.90(m), 7.19(m)
23	Choline	3.20(s), 3.49(m), 4.06(m)
24	Phosphoethanolamine	3.22(m), 3.98(m)
25	Phosphocholine	3.22(s), 3.57(m), 4.17(m)
26	GPC^b^	3.23(s), 3.62(dd), 3.67(m), 3.68(dd), 3.86(m), 3.92(m), 3.95(m), 4.32(m)
27	Glucose	3.23(m), 3.4(m), 3.5(m), 3.53(dd), 3.7(dd), 3.72(dd), 3.78(m), 3.83(m), 3.84(m), 3.94(dd), 4.65(d), 5.23(d)
28	Taurine	3.26(t), 3.43(t)
29	Betaine	3.27(s), 3.89(s)
30	Methanol	3.36(s)
31	myo-Inositol	3.25(t), 3.53(dd), 3.62(t), 4.06(m)
32	Glycine	3.55(s)
33	Ascorbate	3.73(dd), 3.76(dd), 4.02(m), 4.52(d)
34	Uridine	3.8(dd), 3.89(dd), 4.13(m), 4.23(t), 4.36(t), 5.9(d), 5.92(d), 7.88(d)
35	Inosine	3.84(dd), 3.92(dd), 4.27(m), 4.44(m), 4.76(t), 6.11(d), 8.23(s), 8.35(s)
36	Nicotinurate	3.96(m), 7.59(dd), 8.25(m), 8.71(m), 8.94(d)
37	AMP	4.01(m), 4.04(m), 4.38(m), 4.52(dd), 4.79(dd), 6.15(d), 8.26(s), 8.63(s)
38	ATP	4.25(m), 4.29(m), 4.38(m), 4.58(dd), 4.75(dd), 6.16(d), 8.23(s), 8.6(s)
39	ADP	4.20(m), 4.24(m), 4.38(m), 4.58(dd), 4.76(dd), 6.16(d), 8.26(s), 8.54(s)
40	Hypoxanthine	8.16(s), 8.19(s)
41	Formate	8.45(s)

a Multiplicity for ^1^H resonances: s, singlet; d, doublet; t, triplet; q, quartet; m, multiplet; dd, doublet of doublet. Peak multiplicities were extracted from spectral deconvolution of selected samples using Chenomx.

b Abbreviations: GPC, glycerophosphocholine; AMP, Adenosine monophosphate; ATP, Adenosine triphosphate; ADP, Adenosine diphosphate.

**Table 2:** Cigarette smoke and/or obesity induced metabolic changes in lung tissue extracts.

Key	Metabolites	Correlation Coefficients for		
		Model A	Model B	Model C
25	Phosphocholine			-0.896
34	Uridine	0.942	0.867	
35	Inosine	0.892	0.766	
37	AMP	-0.937	-0.843	
38	ATP	-0.921	-0.932	
39	ADP	-0.925	-0.858	
*	Unassigned (δ 3.12)			-0.864
*	Unassigned (δ 3.28)			-0.855
*	Unassigned (δ 5.95)	-0.881	-0.883	
*	Unassigned (δ 6.02)	-0.880	-0.868	
*	Unassigned (δ 6.79)			-0.897

**Table 3:** Two way ANOVA table for GPC/PC ratio.

Variation^a^	Sum of squares	Degree of freedom	Mean sum of squares	*F*-ratio	*p* value
‘Within’	17.07227	28	0.609724		
‘Between’	6.553769	3	2.18459	3.5829	0.026
Class type 1	2.888233	1	2.888233	4.7370	0.038
Class type 2	0.28282	1	0.28282	0.4638	0.501
Interaction	3.382716	1	3.382716	5.5479	0.026

a Variance of the data set is divided into two parts, i.e. within group variance and between group variance. The between group variance is composed of three subparts, including variation caused by obesity (Class type 1), cigarette smoke (Class type 2), and the synergy between these two factors (Interaction).
