# Supplementary material for: Metabolite Signatures in Hydrophilic Extracts of Mouse Lungs Exposed to Cigarette Smoke Revealed by 1H NMR Metabolomics Investigation
Source: Metabolomics (Los Angel). Author manuscript; Available in PMC 2015 Nov 23. (PMC4655886; doi:10.4172/2153-0769.1000143)
Supplement: supporting information [file NIHMS713903-supplement-supporting_information.docx]

Supplementary Information

Metabolite Signatures in Hydrophilic Extracts of Mouse Lungs Exposed to Cigarette Smoke Revealed By ^1^H NMR Metabolomics Investigation

Jian Zhi Hu^*,†^, Xuan Wang^†,‡,$^, Ju Feng^†,$^, Bobbie-Jo Webb-Robertson^†^, Katrina M Waters^†^, Susan C Tilton^†^, Joel G Pounds^†^, Richard A Corley^†^, Maili Liu^‡^, and Mary Hu^†^

^†^ Pacific Northwest National Laboratory, Richland, WA 99352, USA

^‡^ State Key Laboratory of Magnetic Resonance and Atomic and Molecular Physics, Wuhan Institute of Physics and Mathematics, the Chinese Academy of Sciences, Wuhan, 430071, PR China

^$^ These authors have equal contributions to this work.

* To whom correspondence should be addressed:

Jian Zhi Hu; Email: [Jianzhi.Hu@pnnl.gov](mailto:Jianzhi.Hu@pnnl.gov); Phone: (509) 371-6544; Fax: (509) 371-6546


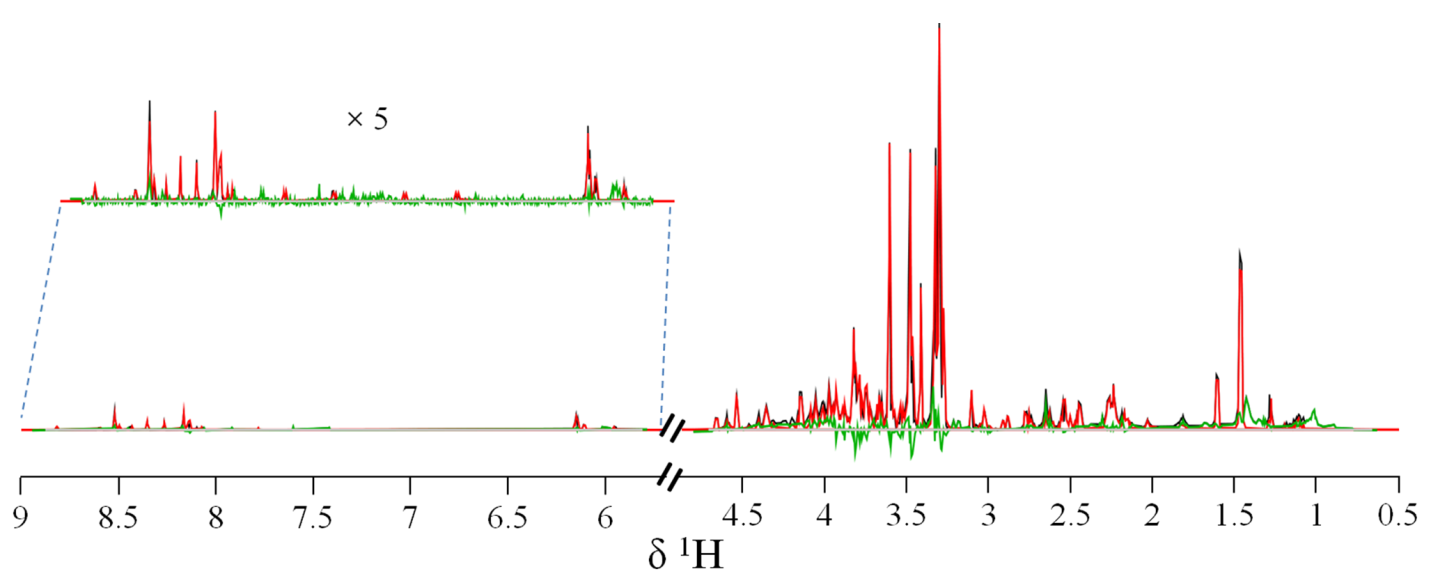


**Figure S1.** Deconvolution of a representative spectrum using Chenomx. The low field regions were vertically expanded 5 times compared with the high field regions of the spectrum. Black line is the original spectrum while red line shows the fitted spectrum. Green line indicates the fitting error. Spectral deconvolution of selected samples by Chenomx is only used for peak assignment and qualitative analysis.


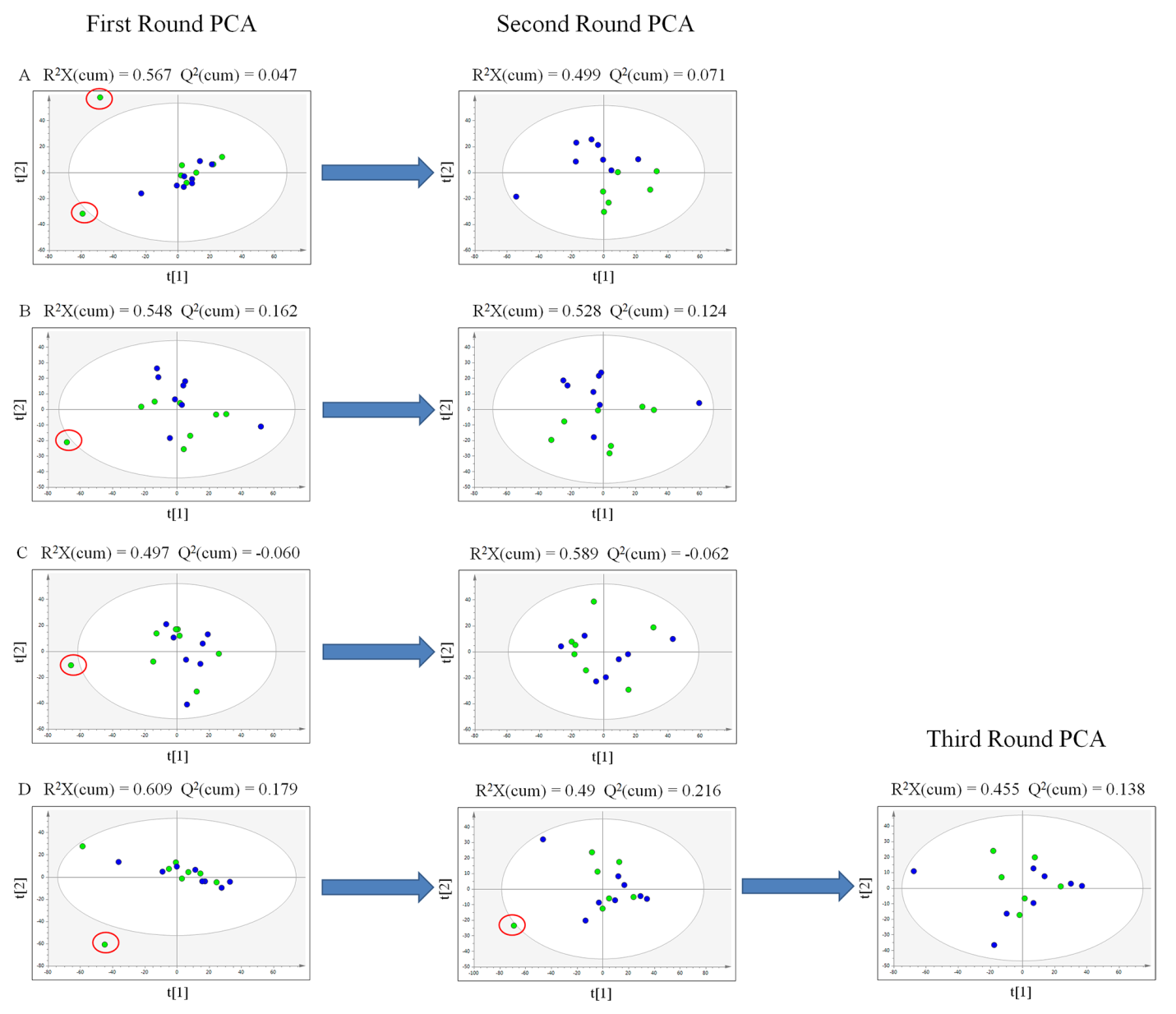


**Figure S2.** PCA scores plots derived from ^1^H NMR data of lung tissue extracts from the control (green dots) and the experimental (blue dots) groups: A, RW-SC (control) *vs* RW-MS (experimental); B, OB-SC (control) *vs* OB-MS (experimental); C, RW-MS (control) *vs* OB-MS (experimental); D, RW-SC (control) *vs* OB-SC (experimental). Samples lying outside the ellipse (Hotelling’s *T*^2^ test, 95%) were regarded as outliers. Outliers (specified by the red cycles) detected in the first (and second in D) round PCA were excluded and then the second (and third in D) round PCA was conducted. If an outlier is found, all the data from that sample were excluded from further analysis.

**Table S1**

Design for two way ANOVA of GPC/PC ratios in the four different treated groups.

|  | | Class type 2  (Control *vs* Cigarette smoke) | |
| --- | --- | --- | --- |
|  | | SC  (Control) | MS  (Cigarette smoke) |
| Class type 1  (Regular weight *vs* Obesity) | RW  (Regular weight) | 4.859813 | 4.918919 |
|  |  | 3.551181 | 6.066667 |
|  |  | 4.292683 | 4.72381 |
|  |  | 3.637097 | 3.542683 |
|  |  | 3.934066 | 3.978102 |
|  |  | 4.067961 | 3.540541 |
|  |  | 4 | 4.337662 |
|  |  | 4.069444 | 3.64 |
|  | OB  (Obesity) | 5.784314 | 8.173913 |
|  |  | 7.386364 | 6.348214 |
|  |  | 7.283951 | 6.084211 |
|  |  | 6.243243 | 7.747253 |
|  |  | 5.069767 | 8.142857 |
|  |  | 5.95 | 6.744186 |
|  |  | 5.098592 | 6.928571 |
|  |  | 5.142857 | 7.487179 |
